# Minimally invasive surgery is feasible after preoperative chemotherapy for stage IV gastric cancer

**DOI:** 10.1002/ags3.12343

**Published:** 2020-05-29

**Authors:** Kazuyoshi Yamamoto, Takeshi Omori, Hisashi Hara, Naoki Shinno, Keijiro Sugimura, Hiroshi Miyata, Hidenori Takahashi, Yoshiyuki Fujiwara, Masayuki Ohue, Masahiko Yano

**Affiliations:** ^1^ Department of Gastroenterological Surgery Osaka International Cancer Institute Osaka Japan

**Keywords:** conversion surgery, gastric cancer, laparoscopic, minimally invasive surgery, robotic

## Abstract

**Aim:**

To elucidate the safety and feasibility of minimally invasive surgery (MIS) as conversion surgery after chemotherapy for stage IV gastric cancer, we compared the background characteristics and clinical courses of patients who underwent open conversion surgery (open group) versus MIS (MIS group).

**Methods:**

We included 94 consecutive patients with stage IV gastric cancer who received chemotherapy followed by conversion surgery gastric resection from January 2011 to October 2019 at the Osaka International Cancer Institute in this analysis.

**Results:**

The open group included more patients who had macroscopic peritoneal metastasis and required splenectomy. However, other background characteristics, including preoperative chemotherapy duration, were comparable. The MIS group had significantly longer operative time (266 vs 339 minutes, *P *= .0039) and less operative blood loss (520 vs 10 mL, *P *< .0001). The incidence of postoperative complication of Clavien‐Dindo grade II or higher was non‐significantly lower (24.5% vs 9.8%, *P *= .058) and length of postoperative hospital stay was significantly shorter in the MIS group (12 vs 8 days, *P *< .0001). Even though the open group included more patients with more advanced (ypT4a or higher, or N3) disease, the MIS group had better recurrence free survival and overall survival (OS). Multivariate analysis revealed that N status (hazard ratio [HR], 4.39; 95% confidence interval [CI], 2.18‐12.26; *P *< .0001) and T status (2.11; 1.05‐4.36; *P *= .036) were independent prognostic factors for OS. MIS was not a negative prognostic factor for OS (HR, 0.44; 95% CI, 0.15‐1.10; *P = *.081).

**Conclusion:**

MIS can be safely performed as conversion surgery following chemotherapy for stage IV gastric cancer.

## Graphical abstract

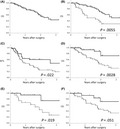

## 1 INTRODUCTION

Gastric cancer is the third most common cause of cancer death throughout the world.^1^ Pathological tumor staging is the most important prognostic determinant for patients with gastric cancer.^2^ In particular, the prognosis of stage IV gastric cancer remains dismal,^3^ despite recent improvements in cancer diagnosis and multimodal treatment. Therefore, a new approach for stage IV gastric cancer is needed to achieve further improvements in gastric cancer treatment.

Conversion surgery for gastric cancer was defined by Yoshida et al as surgical treatment aiming at R0 resection after chemotherapy for tumors that were originally unresectable or marginally resectable for technical or oncological reasons.^4^, ^5^ Conversion surgery has received much attention recently from surgical oncologists because favorable treatment outcomes have been obtained in some cases initially diagnosed as stage IV gastric cancer.^6^, ^7^, ^8^

However, several challenging issues regarding conversion surgery for gastric cancer remain, such as (a) the optimal chemotherapy regimen, (b) optimal duration of preoperative chemotherapy, (c) optimal approach and procedure for conversion surgery, and (d) recommended postoperative chemotherapy after conversion surgery.

In terms of the optimal approach and procedure, minimally invasive surgery (MIS), such as laparoscopic gastrectomy and robotic gastrectomy, has been recognized as a good treatment option for early gastric cancer that is associated with lower postoperative complication rates, less pain, and early recovery.^9^, ^10^, ^11^ Patients with more advanced cancer have also benefited from MIS with comparable postoperative morbidity^12^, ^13^, ^14^, ^15^ and long‐term outcomes^16^ as with the conventional open approach. However, there was no reports to elucidate the effectiveness of MIS as conversion surgery as surgical treatment following chemotherapy for stage IV gastric cancer. The purpose of this study was to evaluate the safety and clinical impact of MIS as conversion surgery after chemotherapy for stage IV gastric cancer.

## 2 METHODS

### 2.1 Patients and preoperative data

A total of 94 consecutive patients with stage IV gastric cancer who received chemotherapy followed by conversion gastric resection from January 2011 to October 2019 at the Osaka International Cancer Institute were included in this analysis. To evaluate the safety and feasibility of MIS, we compared the background characteristics, postoperative clinical course, and survival outcome in the open versus MIS groups. All data were extracted from our prospectively collected database and individual patient medical records. Cancer staging was based on the Japanese Classification of Gastric Carcinoma, third English edition.^17^ Enrolled patients were classified into four categories according to the Yoshida's classification system4 based on the presence or absence of macroscopic peritoneal dissemination and non‐curable metastasis. Patients who were categorized into category 1 received preoperative chemotherapy as neoadjuvant setting, because these tumors were regarded as marginally resectable before treatment. This cohort study was approved by the Human Ethics Review Committee of the Osaka International Cancer Institute (Protocol ID 1608169091).

### 2.2 Preoperative chemotherapy

All 94 patients in this study originally had advanced gastric cancer with peritoneal, hepatic, or distant metastases. They all received preoperative chemotherapy regimens, which were divided into the following three groups: (a) triplet regimen, (b) platinum‐based doublet ± trastuzumab, and (c) regimens that contained intraperitoneal (IP) chemotherapy.

### 2.3 Surgery

When tumor response was observed with computed tomography (CT), which was performed after every two cycles of chemotherapy, curative surgery was attempted. The surgical procedure and type of lymph node dissection used for conversion surgery depended on the site of primary tumor and curability. For R0 resection, para‐aortic lymph node dissection (D3) or partial hepatectomy was attempted if the metastatic tumor was still detected with preoperative CT. Regarding surgical approach, all conversion surgeries following chemotherapy for stage IV gastric cancer were performed via the open approach until 2013. MIS was first used in 2014. The proportion of conversion surgeries performed as MIS increased each year; more than two‐thirds of patients who underwent conversion surgery after 2018 received MIS. Surgeons certified by the Japanese Society for Endoscopic Surgery according to the Endoscopic Surgical Skill Qualification System participated in each conversion surgery of both groups. Postoperative complications were graded according to the Clavien‐Dindo (CD) classification system.^18^ Complications were defined as those that were CD grade II or higher. Complications that were Grade IIIa or higher were considered severe complications.

### 2.4 Postoperative chemotherapy and follow‐up

Patients who underwent conversion surgery gastrectomy following chemotherapy for stage IV gastric cancer received postoperative chemotherapy using S‐1, platinum‐based doublet regimen or taxane depending on the patient's condition and cancer staging until tumor relapse was diagnosed. Follow‐up evaluation consisted of physical examination; blood tests for carcinoembryonic antigen, carbohydrate antigen 19‐9, and carbohydrate antigen 125; and CT. Follow‐up examinations were performed every 3 months.

### 2.5 Statistical analysis

This was a single‐center retrospective observational study. Continuous variables were expressed as medians (range). The χ^2^ test or Fisher's exact test was used to compare categorical variables. The Mann‐Whitney U test was used to compare continuous variables. Univariate and multivariate logistic regression were performed. Recurrence‐free survival (RFS) was defined as the time from conversion surgery to first evidence of clinical recurrence or regrowth of gastric cancer. Survival curves for RFS and overall survival (OS) were estimated using the Kaplan‐Meier method and compared using the log‐rank test. Cox proportional hazard models were used in univariate and multivariate analyses of OS and variables which *P* values were less than 0.1 in univariate analysis were selected to put in the multivariate analysis.

*P* values <.05 were considered statistically significant. Statistical analysis was conducted using JMP^®^ software 14.0 (SAS Institute).

## 3 RESULTS

### 3.1 Patient background characteristics

Overall, median age was 66 (25‐84) years. There were 61 men and 33 women. Fifty‐five patients originally had peritoneal metastasis, 11 patients had liver metastasis, and 31 patients had other distant metastasis, which included para‐aortic lymph node metastasis. There were 38 patients with Yoshida category 1 disease, 8 with category 2, 44 with category 3, and 4 with category 4.

### 3.2 Preoperative chemotherapy

Preoperative chemotherapy regimens and durations were summarized in Table 1. There was only one triplet regimen, docetaxel/cisplatin/S‐1 (DCS).^19^ Platinum‐based doublet ± trastuzumab regimens consisted of S‐1/oxaliplatin (SOX),^20^ S‐1/cisplatin (SP),^21^ S‐1/cisplatin/trastuzumab (SP‐Her),^22^ capecitabine/oxaliplatin (XELOX),^23^ capecitabine/oxaliplatin/trastuzumab (XELOX‐Her),^24^ capecitabine/cisplatin (XP), and capecitabine/cisplatin/trastuzumab (XP‐Her).^25^ All patients who received an IP‐containing regimen (S‐1/paclitaxel/IP paclitaxel), participated in a multicenter clinical trial.^26^

**Table 1:** Preoperative chemotherapy regimens and duration (n = 94)

Regimen	
Triplet	1
DCS	1
Platinum‐based doublet ± trastuzumab	75
SOX	32
SP	16
SP‐Her	5
XELOX	7
XELOX‐Her	7
XP	5
XP‐Her	3
IP‐containing	18

Duration	
Median (range), months	3.3 (1.2‐82.1)
<2	8
2‐3.9	52
4‐5.9	15
≥6	19

Abbreviations: DCS, docetaxel/cisplatin/S‐1^19^; IP, intraperitoneal chemotherapy (S‐1/paclitaxel/IP paclitaxel)^26^; SOX, S‐1/oxaliplatin^20^; SP, S‐1/cisplatin^21^; SP‐Her, S‐1/cisplatin/trastuzumab^22^; XELOX, capecitabine/oxaliplatin^23^; XELOX‐Her, capecitabine/oxaliplatin/trastuzumab^24^; XP, capecitabine/cisplatin^25^; XP‐Her, capecitabine/cisplatin/trastuzumab^25^.

### 3.3 Background characteristics and operative factors of the open and MIS groups

Conversion surgery Gastrectomy following chemotherapy for stage IV gastric cancer was performed using the open approach in 53 patients (open group) and the MIS approach in 41 patients (MIS group). The MIS group included 29 patients who underwent laparoscopic gastrectomy and 12 who underwent robotic gastrectomy. We compared the background characteristics, postoperative clinical course, and survival outcome of the open and MIS groups. Background characteristics and surgical data of the open and MIS groups were described in Table 2.

**Table 2:** Background characteristics and operative factors in the open and MIS groups

	Open (n = 53)	MIS (n = 41)	*P* value
Age (y)	66 (25‐80)	65 (35‐84)	.91
Gender, n (%)			
Men	35 (66.0)	26 (63.4)	.79
Women	18 (34.0)	15 (36.6)	
BMI (kg/m^2^)	22.3 (13.7‐31.1)	22.2 (16.2‐35.7)	.57
Location (U/M/L)	10/26/17	12/21/8	.29
Macroscopic type (Borrmann 2/3/4)	17/22/14	13/19/9	.85
Lauren type (Intestinal/Diffuse)	17/36	21/20	.061
Yoshida's classification, n (%)			
C1	17 (32.0%)	21 (51.2%)	.0024
C2	1 (1.9%)	7 (17.1%)	
C3	32 (60.4%)	12 (29.3%)	
C4	3 (5.7%)	1 (2.4%)	
Chemotherapy regimen			
Triplet or doublet	39 (73.6%)	37 (90.2%)	.036
IP‐containing	14 (26.4%)	4 (9.8%)	
Duration of chemotherapy (months)	3.4 (1.6‐15.7)	3.2 (1.2‐82.1)	.37
Preoperative albumin level (g/dL)	3.7 (1.9‐4.7)	3.8 (2.4‐4.7)	.34
ASA‐PS, n (%)			
1, 2	49 (92.4)	40 (97.6)	.27
3	4 (7.6)	1 (2.4)	
Procedure, n (%)			
TG	30 (56.6%)	20 (48.8)	.45
Non‐TG	23 (43.4%)	21 (51.2)	
Lymph node dissection, n (%)			
D1+	11 (20.8%)	5 (12.2%)	.12
D2	35 (66.0%)	24 (58.5%)	
D3	7 (13.2%)	12 (29.3%)	
Combined resection (yes (%)/ no)	15 (28.3%)/ 38	9 (22.0%)/ 32	.48
Spleen	8 (15.1%)	1 (2.4%)	.026
Liver	2	4	
Transverse colon	2	1	
Lower esophagus	0	3	
Small intestine	2	0	
Ovary	1	0	
Resectability, n (%)			
R0	36 (67.9%)	28 (68.3%)	.64
R1	12 (22.6%)	7 (17.1%)	
R2	5 (9.4%)	6 (14.6%)	
Operative time (min)	266 (154‐470)	339 (155‐607)	.0039
Operative blood loss (mL)	520 (85‐1555)	10 (0‐430)	<.0001
Open conversion (yes/no)		0/41	

Abbreviations: ASA‐PS, American Society of Anesthesiologists physical status; BMI, body mass index; IP, intraperitoneal chemotherapy; Location (U/M/L), Location (Upper/Middle/Lower); MIS, minimally invasive surgery; non‐TG, non‐total gastrectomy, which includes distal gastrectomy and proximal gastrectomy; TG, total gastrectomy; Triplet or doublet, Triplet or platinum‐based doublet ± trastuzumab.

The open group included more patients with macroscopic peritoneal metastasis classified in Yoshida's category C3 and C4 (*P = .*0024) and patients who received IP‐containing regimen (*P = .*036). Other background characteristics and the duration of preoperative chemotherapy were comparable between the two groups. Surgical characteristics, resection type, extent of lymph node dissection, and R0 rate of the two groups were similar. The rate of combined resection was also similar; however, the rate of splenectomy was significantly higher in the open group (*P = .*026). The MIS group had significantly longer operative time (266 vs 339 minutes; *P = .*0039) and less operative blood loss (520 vs 10 mL, *P *< .0001) than the open group. None of the patients in the MIS group required conversion to open surgery.

### 3.4 Postoperative complications and clinical course

The incidence of postoperative complications of CD grade II or higher was lower in the MIS group than in the open group (24.5% vs 9.8%; *P = .*058) but the difference was not statistically significant. None of the patients in the MIS group developed severe postoperative complications of CD grade III or higher. There was no significant difference in the distribution of complications or their severity between the two groups. Length of hospital stay after conversion surgery was significantly shorter in the MIS group (12 vs 8 days; *P *< .0001). There was no mortality within 90 days after surgery in either group (Table 3).

**Table 3:** Postoperative complications and clinical course

	Open (n = 53)	MIS (n = 41)	*P* value
Postoperative complication CD grade ≥ II (yes(%)/no)	13 (24.5%)/ 40	4 (9.8%)/ 37	.058
Leakage	2 (3.8%)/ 51	0/41	.21
Pancreatic fistula	1 (1.9%)/ 52	1 (2.4%)/ 40	.85
Abdominal abscess	3 (5.7%)/ 50	0/41	.12
Pneumonia	2 (3.8%)/ 51	0/41	.21
Superficial SSI	2 (3.8%)/ 51	1 (2.4%)/ 40	.71
DGE	3 (5.7%)/ 50	2 (4.9%)/ 39	.86
Lymphatic fistula	2 (3.8%)/ 51	0/41	.21
Cholecystitis	1 (1.9%)/ 52	0/41	.38
Anastomotic stenosis	1 (1.9%)/ 52	0/41	.38
Postoperative complications CD grade ≥ III (yes(%)/no)	4 (7.6%)/ 49	0/41	.072
Mortality	0	0	
Median (range) length of hospital stay (days)	12 (6‐100)	8 (6‐15)	<.0001

Abbreviations: CD, Clavien‐Dindo; DGE, delayed gastric emptying; MIS, minimally invasive surgery; SSI, surgical site infection.

### 3.5 Pathological findings and postoperative chemotherapy

Pathological findings, histological evaluation of chemotherapy effects, and postoperative chemotherapy were presented in Table 4. The open group included more aggressive cases with ypT4a or higher or ypN3 disease. Pathological response was similar between the two groups. The pathologic complete response (CR) rate of the open and MIS groups was 7.6% and 14.6%, respectively. The rate of postoperative chemotherapy induction was sufficiently high in both groups (90.6% vs 95.1%; *P = .*39). There were no significant differences in postoperative chemotherapy regimens. Duration from conversion surgery to initiation of postoperative chemotherapy was significantly shorter in the MIS group (39 vs 25 days; *P = .*0008).

**Table 4:** Pathological findings and postoperative chemotherapy

	Open (n = 53)	MIS (n = 41)	*P* value
Pathological findings			
ypT0‐3/T4a‐4b	28/25 (47.2%)	30/11 (26.8%)	.044
ypN0‐2/N3	26/27 (50.9%)	31/10 (24.4%)	.0081
ypM0/M1	30/23 (43.4%)	21/20 (48.8%)	.60
Pathological response (1a/1b/2/3)	37/3/9/4 (7.6%)	20/4/11/6 (14.6%)	.22
Final stage (CR I/II/III/IV)	7/11/12/23	9/7/5/20	.42
Postoperative chemotherapy (yes/no)	48 (90.6%)/5	39 (95.1%)/2	.39
Postoperative chemotherapy regimen			
S‐1 alone	22 (45.8%)	17 (43.6%)	.88
Platinum‐based doublet	24 (50.0%)	21 (53.8%)	
Taxane	2 (4.2%)	1 (2.6%)	
Reason for interruption of postoperative chemotherapy			
CR	1	1	
Delayed recovery	2	0	
Tumor progression	2	1	
Median (range) duration from surgery to postoperative chemotherapy (days)	39 (15‐123)	25 (16‐60)	.0008

Note

Gastric cancer staging and histological evaluation after preoperative chemotherapy was based on the Japanese classification of gastric carcinoma, third English edition.^17^

Abbreviation: CR, complete response; MIS, minimally invasive surgery.

### 3.6 Survival

**Figure 1:**
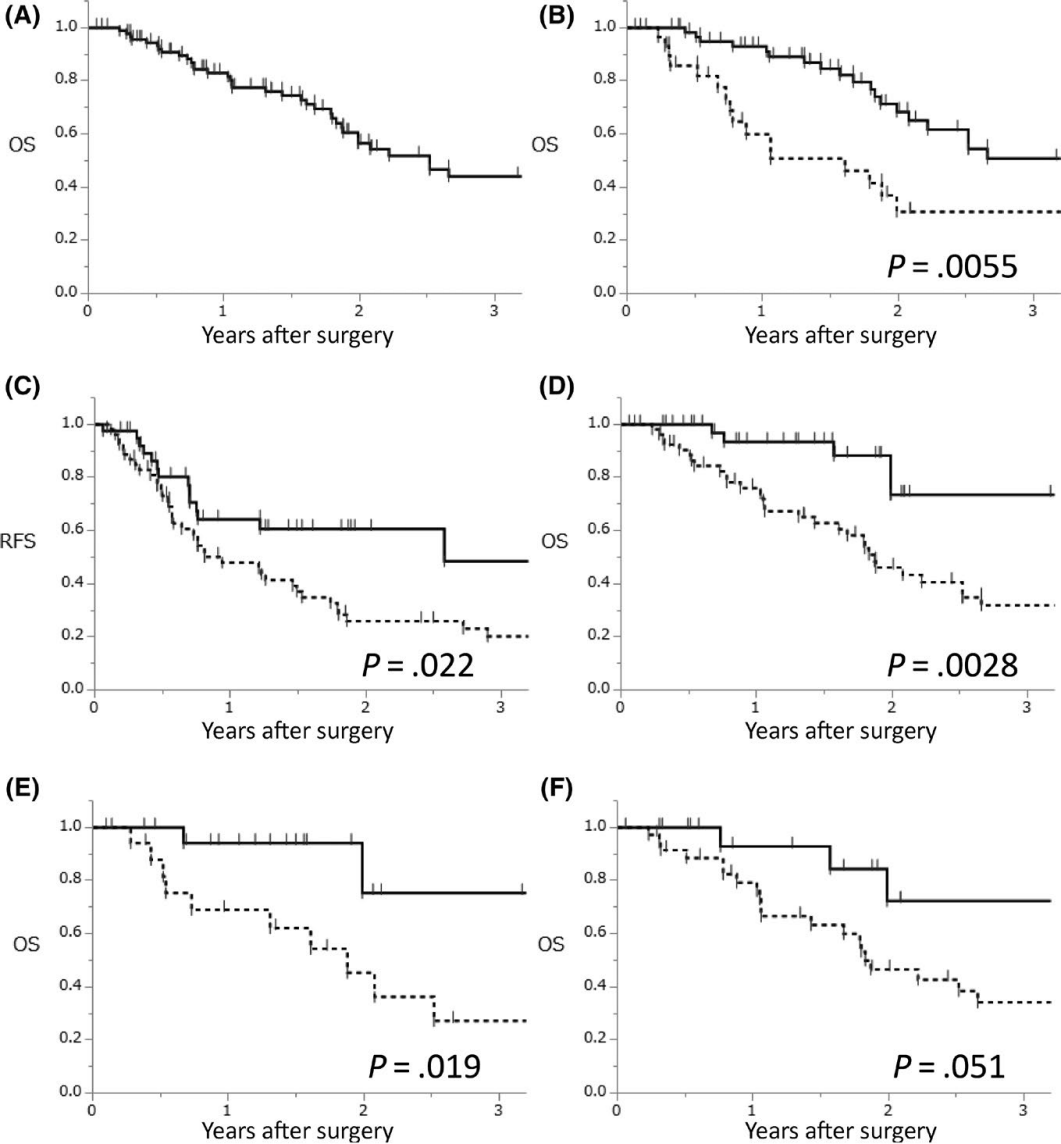
Survival. A, OS after conversion surgery overall (n = 94) MST was 30.2 mo. B, OS after conversion surgery in patients who achieved R0 resection (n = 64) and R1 or R2 resection (n = 30). The solid line indicates the survival curve of patients with R0 resection; MST was 42.4 mo. The dotted line indicates the survival curve of patients with R1 or R2 resection; MST was 19.3 mo. C, RFS after conversion surgery in the open group (n = 53) and the MIS group (n = 41). The solid line indicates the survival curve of the MIS group; median time to recurrence was 31.0 mo. The dotted line indicates the survival curve of the open group; median time to recurrence was 11.3 mo. D, OS after conversion surgery in the open group (n = 53) and the MIS group (n = 41). The solid line indicates the survival curve of MIS group; MST was 52.7 mo. The dotted line indicates the survival curve of the open group; MST was 22.4 mo. E, OS after surgery of category 1 according to the Yoshida's classification in the open group (n = 17) and the MIS group (n = 21). The solid line indicated the survival curve of MIS group; MST was more than 52.6 mo. The dotted line indicated the survival curve of the MIS group; MST was 22.6 mo. F, OS after surgery of category 2‐4 according to the Yoshida's classification in the open group (n = 36) and the MIS group (n = 20). The solid line indicated the survival curve of MIS group; MST was 52.7 mo. The dotted line indicated the survival curve of the MIS group; MST was 22.0 mo. OS, overall survival; RFS, recurrence‐free survival; MIS, minimally invasive surgery; MST, median survival time

Survival analysis was performed after a median observational period of 18 months. Estimated OS of all enrolled patients (n = 94) was shown in Figure 1A. Median survival time (MST) was 30.2 months. As previously reported, patients who achieved R0 resection (n = 64) had better survival outcome than patients with R1 or R2 resection (n = 30) (MST, 42.4 vs 19.3 months; *P = .*0055) (Figure 1B). The MIS group had better RFS (median time to recurrence, 11.3 vs. 31.0 months; *P *= .022) (Figure 1C) and OS compared with the open group (MST, 22.4 vs 52.7 months; *P *= .0028) (Figure 1D). This tendency was maintained even when the patients were separately compared among category 1 (Figure 1E) and category 2‐4 (Figure 1F), and difference was more significant in category 1 in which patients who received preoperative chemotherapy as neoadjuvant setting.

### 3.7 Impact of MIS on OS

To evaluate the impact of MIS on OS in patients who underwent conversion surgery gastrectomy after preoperative chemotherapy for stage IV gastric cancer, we used Cox proportional hazards models to stratify by cancer stage (Table 5). Surgical approach (MIS vs. open), along with resectability (R1or R2 vs R0), T status (ypT4a‐4b vs T0‐3), N status (ypN3 vs N0‐2), M status (ypM1 vs M0), and histological response (CR vs non‐CR) were significant prognostic factors in univariate analyses. In multivariate analysis, N status (hazard ratio [HR], 4.93; 95% confidence interval [CI], 2.18‐12.26; *P *< .0001) and T status (2.11; 1.05‐4.36; *P* = .036) were independent prognostic factors on OS. MIS was not a negative prognostic factor for OS after conversion surgery (HR, 0.44; 95% CI, 0.15‐1.10; *P = *.081).

**Table 5:** Univariate and multivariate analysis of OS after conversion surgery

		n	Univariate			Multivariate		
			HR	95% CI	*P*	HR	95% CI	*P*
Age	≥70 y	25	1.44	0.66‐2.89	.35			
	<70 y	69						
Gender	Male	61	1.04	0.53‐2.14	.92			
	Female	33						
Category	C2‐4	56	1.17	0.60‐2.42	.65			
	C1	38						
Chemotherapy regimen	IP	18	1.95	0.96‐3.79	.066	1.30	0.55‐2.98	.54
	T or D	76						
Approach	MIS	41	0.29	0.11‐0.64	.0016	0.44	0.15‐1.10	.081
	Open	53						
Procedure	TG	50	1.78	0.92‐3.57	.085	0.85	0.39‐1.84	.67
	Non‐TG	44						
Resectability	R1 or R2	30	2.45	1.25‐4.70	.0095	1.84	0.85‐3.95	.12
	R0	64						
Postoperative complications	Yes	17	1.09	0.46‐2.27	.83			
	No	77						
Tumor depth	ypT4a‐4b	36	3.57	1.86‐7.01	.0002	2.11	1.05‐4.36	.036
	ypT0‐3	58						
Lymph node metastasis	ypN3	37	7.54	3.70‐16.46	<.0001	4.93	2.18‐12.26	<.0001
	ypN0‐2	57						
Distant metastasis	ypM1	43	3.05	1.57‐6.20	.0009	1.78	0.85‐3.84	.12
	ypM0	51						
Histological response	CR	10	0.29	0.048‐0.97	.044	0.96	0.22‐6.62	.96
	Non‐CR	84						
Postoperative chemotherapy	Yes	87	0.71	0.25‐2.96	.58			
	No	7						

Note

Gastric cancer staging and histological evaluation after preoperative chemotherapy were based on the Japanese classification of gastric carcinoma, third English edition.^17^

Abbreviations: CI, confidence interval; CR, complete response; HR, hazard ratio; IP, intraperitoneal chemotherapy; IP, intraperitoneal chemotherapy; MIS, minimally invasive surgery; OS, overall survival; T or D, Triplet or platinum‐based doublet ± trastuzumab; TG, total gastrectomy.

## 4 DISCUSSION

Recent improvements in systematic chemotherapy, molecular targeting agents,^25^ and immune checkpoint inhibitors^27^ have improved the prognosis of stage IV gastric cancer. However, MST for stage IV gastric cancer remains unsatisfactory, around 13‐16 months.^20^, ^21^, ^25^, ^26^ Conversion surgery for stage IV gastric cancer has led to excellent treatment outcomes in some patients, which has attracted great interest from surgical oncologists.^4^, ^5^, ^6^, ^7^, ^8^

On the other hand, laparoscopic gastrectomy and robotic gastrectomy, referred to as MIS, are accepted as more effective than conventional open surgery and are commonly used for clinical stage I gastric cancer in accordance with recent improvements in technical and instrumental aspects.^9^, ^10^, ^11^ Even for locally advanced gastric cancer, the technical safety of laparoscopic distal gastrectomy (LDG) was shown in a randomized phase II study (JLSSG0901).^12^ A large phase III trial (KLASS‐02‐RCT)^13^ showed that LDG with D2 lymphadenectomy is associated with a lower postoperative complication rate, faster recovery, and less pain than open distal gastrectomy (ODG). Furthermore, the large phase III randomized clinical trial CLASS‐01^16^ demonstrated the non‐inferiority of LDG in terms of 3‐year disease‐free survival compared to ODG. Moreover, MIS also offers benefits in gastrectomy for locally advanced gastric cancer after neoadjuvant chemotherapy, such as better postoperative safety and adjuvant chemotherapy tolerance compared with conventional open surgery.^28^, ^29^

At the Osaka International Cancer Institute, MIS has been employed for locally advanced gastric cancer in a clinical trial setting. It was first adopted in 2014 as conversion surgery surgical treatment following chemotherapy for stage IV gastric cancer with adequate explanation and informed consent about surgical and oncological risks. Subsequently, the proportion of conversion surgeries performed as MIS has increased each year. We evaluated the safety and clinical impact of MIS for conversion surgery in this study.

This study showed that MIS as conversion surgery had non‐significantly lower surgical morbidity and shorter postoperative hospital stay compared to open surgeries. The duration from surgery to initiation of postoperative chemotherapy was significantly shorter in the MIS group than in the open group. Furthermore, RFS and OS of MIS group was at least, comparable to open group. These results were consistent with the results of large‐scale randomized controlled trials comparing LDG and conventional ODG in locally advanced gastric cancer, which formed the basis for using MIS as conversion surgery following chemotherapy for stage IV gastric cancer.

This study has several limitations. First, this observational study was conducted in a retrospective manner. There was substantial difference in background characteristics, surgical factors, and pathological findings between the open and MIS groups. The open group included more patients with category 3 and 4 disease who had macroscopic peritoneal dissemination before chemotherapy. Even though final staging was comparable between the two groups, the proportion of patients with ypT4a or higher and ypN3 disease was higher in the open group compared with the MIS group. Combined resection of the spleen was performed more often in the open group. The incidence of postoperative complications of CD grade II or higher also tended to be lower in the MIS group than in the open group among patients who did not undergo splenectomy (22.2% vs 10.0%; *P *= .12). Second, the median follow‐up periods of 18 months overall and 17 months in the MIS group were insufficient for evaluating the long‐term effects of MIS versus open surgery. Therefore, longer follow‐up will be necessary to confirm the results regarding survival outcome. Third, subjective parameters such as pain score, patient satisfaction, and quality of life to evaluate other potential benefits of MIS were not included in this study. Nevertheless, this is the first report regarding the feasibility of MIS as conversion surgery following chemotherapy for stage IV gastric cancer. The prognosis of stage IV gastric cancer remains dismal and the main treatment strategy for stage IV gastric cancer is chemotherapy, not surgery. MIS might be recommended as conversion surgery following chemotherapy to minimize interruption of chemotherapy because in this study, the incidence of postoperative complications was low, and the length of hospital stay and duration from surgery to initiation of postoperative chemotherapy were shorter in the MIS group. Therefore, a well‐designed randomized controlled trial comparing MIS and conventional open gastrectomy with adequate follow‐up will be necessary to confirm our results.

In conclusion, our results suggest that MIS can be safely performed as conversion surgery following after chemotherapy for stage IV gastric cancer.

## CONFLICT OF INTEREST

Authors declare no conflicts of interest for this article.

## DISCLOSURE OF ETHICAL STATEMENTS

This cohort study was approved by the Human Ethics Review Committee of the Osaka International Cancer Institute (Protocol ID 1608169091).
